# Pyrolysis Reactions of (2-Chloroethyl)benzene

**DOI:** 10.1021/acs.jpca.5c03721

**Published:** 2025-10-03

**Authors:** Mia Jarrell, Tess Courtney, Khaled El-Shazly, David Kapp, Andrew Fields, Alexis Bowles, Laura R. McCunn

**Affiliations:** Department of Chemistry, 4034 Marshall University 1 John Marshall Drive, Huntington, West Virginia 25755, United States

## Abstract

Pyrolysis of polyvinyl chloride (PVC) is considered an alternative to traditional, mechanical methods of recycling. However, there is insufficient research conducted on the thermal decomposition pathways of PVC, particularly the fate of chlorinated hydrocarbons generated during the chemical recycling process. One significant product from the pyrolysis of PVC is (2-chloroethyl)­benzene. Using a hyperthermal tubular reactor and matrix-isolation FTIR techniques, the pyrolysis products of gas-phase (2-chloroethyl)­benzene were identified. Following pyrolysis at 1400 K, the FTIR spectra indicated the formation of HCl, styrene, phenylacetylene, benzene, vinylacetylene, acetylene, propyne, ethylene, and propargyl radical.

## Graphical abstract

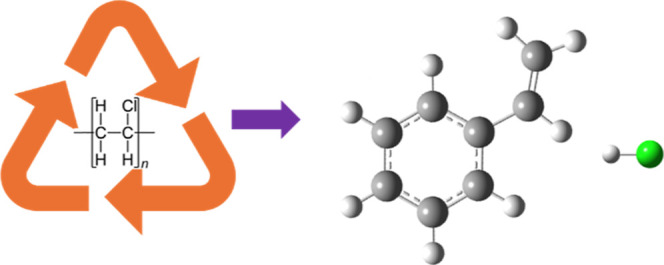

## Introduction

Polyvinyl chloride (PVC) is a synthetic thermoplastic material commonly used in building materials. In recent years, it has ranked among the top three major polymers for global plastics production^1–3^ and among the top five in market share and industrial applications.^4^ Recent research, however, documents many unfavorable health^5–10^ and environmental^11–13^ outcomes associated with its use and waste management. The increasing production rate of plastics and the low recycling rate have resulted in an enormous amount of global waste.^13^ Plastics like PVC are nonbiodegradable and tend to persist in the environment. PVC is particularly problematic among plastics because it can leach toxic chlorinated compounds into the environment and the food chain.^4^ These concerns and health hazards emphasize the need for a clean and economically viable recycling method.

PVC is most commonly broken down through mechanical recycling methods. The mechanical recycling process shreds, grinds, or mills the plastic and then washes and dries the processed material to be sorted and melted into new PVC product. This would be a sustainable technique for reducing PVC waste if the material to be recycled had low contamination and could be recycled in the same area where the waste was produced. Unfortunately, current plastic recycling strategies require expensive sorting and transportation for processing. Furthermore, the recycled product has a lower purity and durability than nascent plastic, reducing its commercial value. Thus, chemical recycling is receiving increasing attention as a more sustainable and effective approach to manage PVC waste.^14,15^ Chemical recycling is the process of converting plastic polymers into smaller molecules that can be refined into valuable chemical feedstocks, alternative fuels, and even reagents for the synthesis of new plastics.^16–18^ This method can be used on mixed plastics, eliminating the need for expensive sorting processes, and is generally more efficient than mechanical recycling.^3,19^ Pyrolysis or catalysts are often employed to breakdown plastic polymers in chemical recycling.

The pyrolysis of PVC primarily releases HCl, aromatics, hydrocarbons, and chlorinated hydrocarbons.^20–22^ The chlorinated hydrocarbons are of special concern in the chemical recycling of PVC because they are toxic, corrosive, and have limited industrial value.^2,3,23^ One common example of a chlorinated hydrocarbon derived from the pyrolysis of PVC is (2-chloroethyl)­benzene, shown in Figure 1. Several experiments have observed this compound during PVC pyrolysis under various conditions. In 1999, Miranda et al.^24^ used a bench scale batch reactor under vacuum to perform PVC pyrolysis at various temperatures. Approximately 200 g of the PVC powder sample was loaded into the reactor and externally heated, ranging from temperatures of 220 to 520 °C. The gas-phase products collected at the end of the pyrolysis reaction were analyzed by a gas chromatograph. The data revealed that chlorinated hydrocarbons were formed, including chlorobenzene, 1-chloro-2-ethylbenzene, benzyl chloride, and (2-chloroethyl)­benzene, appearing as the temperature increased. The weight percents, on the pyrolysis oil basis, of these chlorinated aromatics varied with pyrolysis temperature but ranged from 0.01% to 3.21%, with benzyl chloride as the most abundant species. Aracil et al.^25^ performed PVC pyrolysis and combustion experiments over 500–1000 °C using a horizontal quartz tube-type reactor that was placed inside a furnace. The PVC powder sample traveled through the furnace where decomposition occurred, and its products were collected at the end of the reactor tube. It was found that (2-chloroethyl)­benzene was among the semivolatile products of PVC at 500 °C, detected at a level of 64 mg/kg sample. Under those same conditions, only 1-chloro-dodecane was detected at a higher amount, 173 mg/kg. Four other chlorinated hydrocarbons (C9–C16) were detected at significantly lower levels.

**Figure 1:**
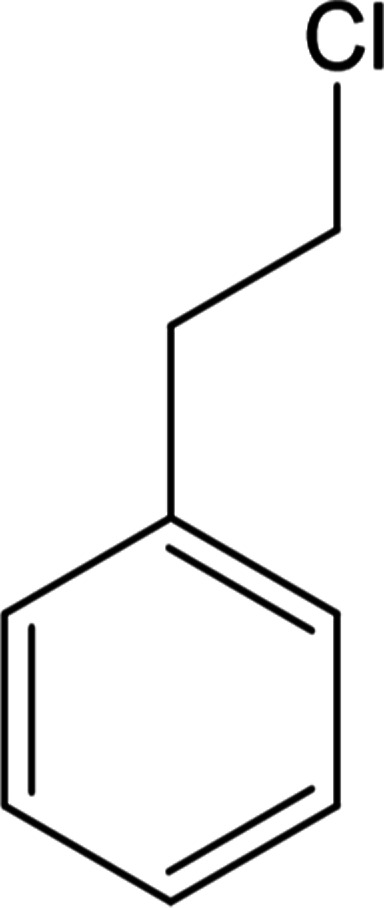
(2-Chloroethyl)­benzene.

There has been much research focusing on thermal decomposition pathways for PVC, but limited work has focused on the thermal decomposition pathway of the individual chlorinated hydrocarbons generated during that process. The simplest aromatic chlorides, chlorobenzene and benzyl chloride, have been studied by pyrolytic techniques,^26,27^ so the unstudied and slightly more complex (2-chloroethyl)­benzene is a natural choice for a pyrolysis study. Understanding the high-temperature reactions of (2-chloroethyl)­benzene will facilitate the development of a pyrolysis mechanism and also the development of methods to manage undesired byproducts of the PVC chemical recycling process.

The purpose of this work is to study the pyrolysis of (2-chloroethyl)­benzene. Observation of the pyrolysis products of (2-chloroethyl)­benzene was accomplished by using a pulsed, hyperthermal, small tubular reactor coupled with a matrix-isolation Fourier transform infrared spectrometer. Pyrolysis temperatures ranging from 900 to 1400 K were employed to observe the effect of temperature on reaction pathways. The application of the matrix-isolation Fourier transform infrared spectrometer allowed the produced species to be captured and isolated in a low-temperature argon matrix and then characterized by spectroscopic analysis. The sample residence time in the reactor was fairly short, on the order of 100 μs, and thus, the observed products provide insight into early steps in the pyrolysis mechanism.

## Methods

The pyrolysis of (2-chloroethyl)­benzene was accomplished by a pulsed hyperthermal tubular reactor^28^ coupled with a matrix-isolation Fourier transform infrared spectrometer. Argon gas (750 Torr) was bubbled through the liquid sample (0.5 Torr of vapor pressure), yielding a 0.07% mixture. The dilute mixture expanded through the orifice of a Parker General Valve Series 9 pulsed valve into a resistively heated silicon carbide (SiC) tube with a length of 3.8 cm and 1 mm inner diameter. The temperature of the pyrolysis tube was manually controlled by a variable transformer and monitored by a type C thermocouple connected to a Love Controls Series 16A temperature controller. The pulsed valve operated with an on-time of 200 μs and an off-time of 20 ms, yielding a gas flow rate of 3 mmol/h. Recent studies^29,30^ of similar hyperthermal reactors thoroughly describe how the temperature profile and flow dynamics are affected by experimental conditions and conclude that these reactors generally have flow in the laminar domain and tend to suppress secondary reactions at low concentrations. The pyrolysis products were sprayed from the SiC tube onto a cold cesium iodide (CsI) window which was mounted inside an evacuated cryostat with a pressure of 1 × 10^–6^ Torr. The CsI window was cooled by a closed-cycle Sumitomo Heavy Industrial Cryocooler Model SRDK-101 and adjusted to 15 K by a LakeShore 331 temperature controller during deposition. The low temperature allowed the pyrolysis products to be captured and frozen in an argon matrix. Following 3 hours of pyrolysis and deposition, the window in the cryostat was cooled to 4 K for FTIR spectral analysis. A total of 128 scans were recorded using a Bruker Vertex 70 Fourier-transform infrared spectrometer under dry air purge and 0.5 cm^–1^ resolution.

Reactions of (2-chloroethyl)­benzene were investigated computationally using Gaussian 09 at the B3LYP/6-311G++(d,p) level of theory.^31–33^ Zero-point corrected energies of the reactant, intermediates, transition states, and products were determined by optimization and frequency calculations. Transition states were confirmed by the intrinsic reaction coordinate calculations.

## Results and Discussion

The spectra collected following pyrolysis at temperatures from 900 to 1400 K were compared to a spectrum of unheated (2-chloroethyl)­benzene in order to identify bands that are evidence of pyrolysis products. Figure 2 shows these spectra stacked for comparison, revealing that pyrolysis above 1100 K yielded nearly complete conversion to the product. This is evidenced by the band around 725 cm^–1^ that appears smaller in the spectrum collected for 900 K pyrolysis and is almost nonexistent in the spectra collected for 1300 and 1400 K. It should be noted that some vibrational bands appear to persist in the spectra for the pyrolysis temperatures in Figure 2, such as the band around 695 cm^–1^, because some of the products of pyrolysis have vibrational bands that overlap those of the parent molecule. The products and intermediates from the pyrolysis of (2-chloroethyl)­benzene were identified by comparison to literature vibrational frequencies of matrix-isolated species under similar conditions. HCl was one of the major pyrolysis products recorded. Other products include styrene, phenylacetylene, benzene, vinylacetylene, acetylene, propyne, ethylene, and the propargyl radical. For the sake of convenience, the spectrum collected following pyrolysis at 1400 K will be used in the following discussion of product assignments because it yielded the best signal-to-noise ratio and also included all of the products that were observed following pyrolysis at lower temperatures. Figures that report “Relative Absorbance” contain stacked spectra with absorbances that are directly comparable.

**Figure 2:**
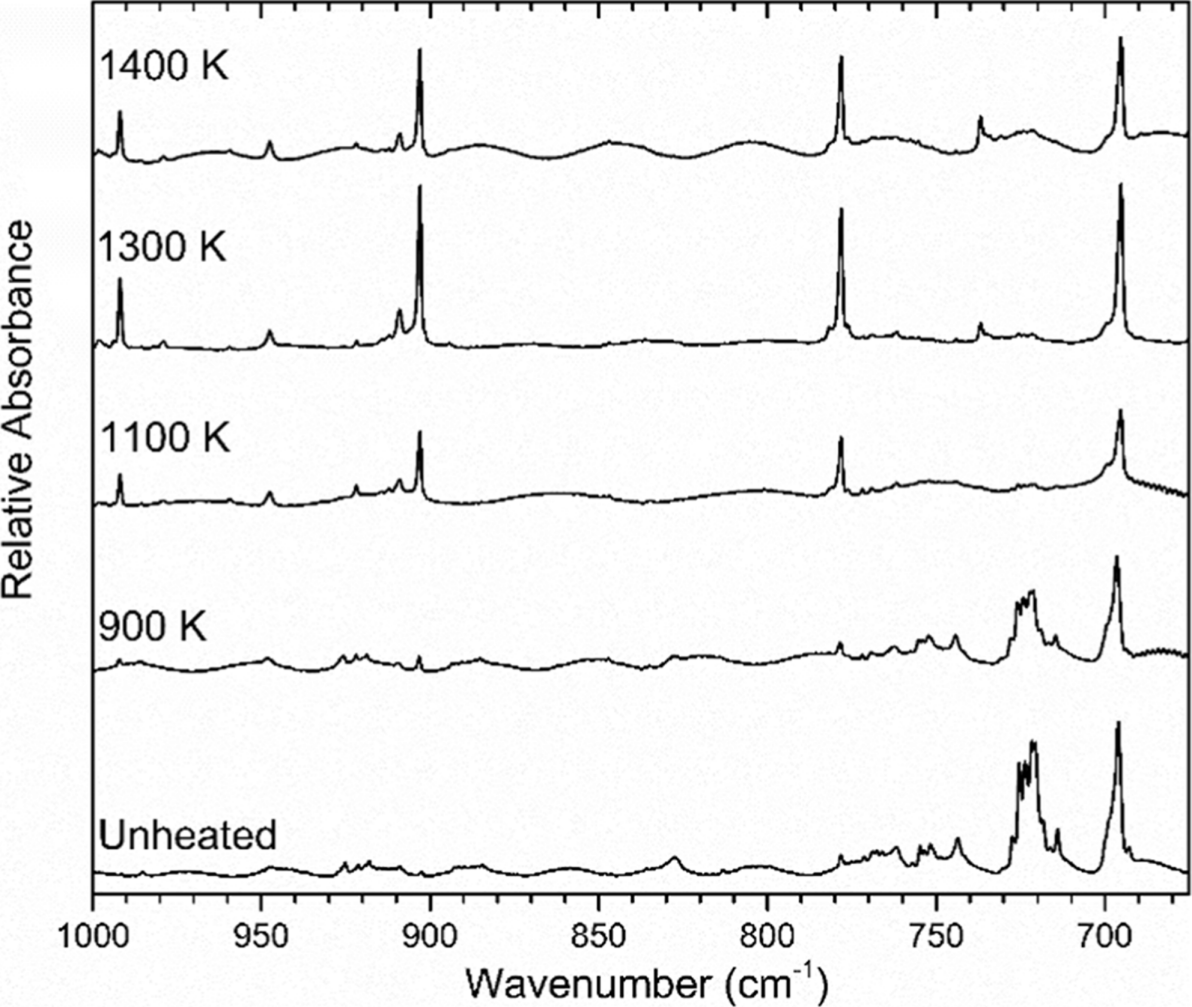
A temperature study of (2-chloroethyl)­benzene, comparing the argon-matrix FTIR spectrum of an unheated sample to those collected following pyrolysis at temperatures ranging from 900 to 1400 K. All samples were 0.07% mixtures in argon.

It is evident that HCl is produced when (2-chloroethyl)­benzene is thermally decomposed. As seen in Figure 3, a strong, sharp band appears in the heated spectrum at 2888 cm^–1^. This agrees with the literature-reported R(0) band of HCl reported at 2888 cm^–1^ by Huang et al.^34^ and Lignell et al.^35^ The H^37^Cl isotopomer adjoins this band at 2885 cm^–1^. There is a very low absorption band at 2871 cm^–1^, corresponding to the Q-band of HCl observed in an argon matrix and reported in several other papers.^36^ There is an even weaker band corresponding to the P(1) band of HCl at 2853 cm^–1^.^37^ There are many other low-absorption bands in the 2875–2750 cm^–1^ region as shown in Figure 3. It is presumed that they are due to HCl multimers or various clusters of HCl with other species present in the product mixture. The HCl·H_2_O band^38^ is present at 2664 cm^–1^. Other possible assignments of cluster bands in Figure 3 include HCl·(H_2_O)_2_ at 2754 cm^–1^,^38^ (HCl)_2_ at 2815 cm^–1^,^36^ and HCl·ethylene at 2753 cm^–1^.^39^ In order to determine whether the spectrum may contain bands for clusters of HCl with prominent pyrolysis products styrene or phenylacetylene, which are discussed below, codeposition experiments were carried out with 1:1:1000 mixtures of HCl/sample/argon for each of styrene and phenylacetylene. In those spectra, there was evidence that the bands in the 2780–2760 cm^–1^ region are likely due to styrene·HCl clusters (Figure S1). There was much weaker evidence for the production of phenylacetylene·HCl clusters and thus it was concluded that other HCl-containing clusters must be involved.

**Figure 3:**
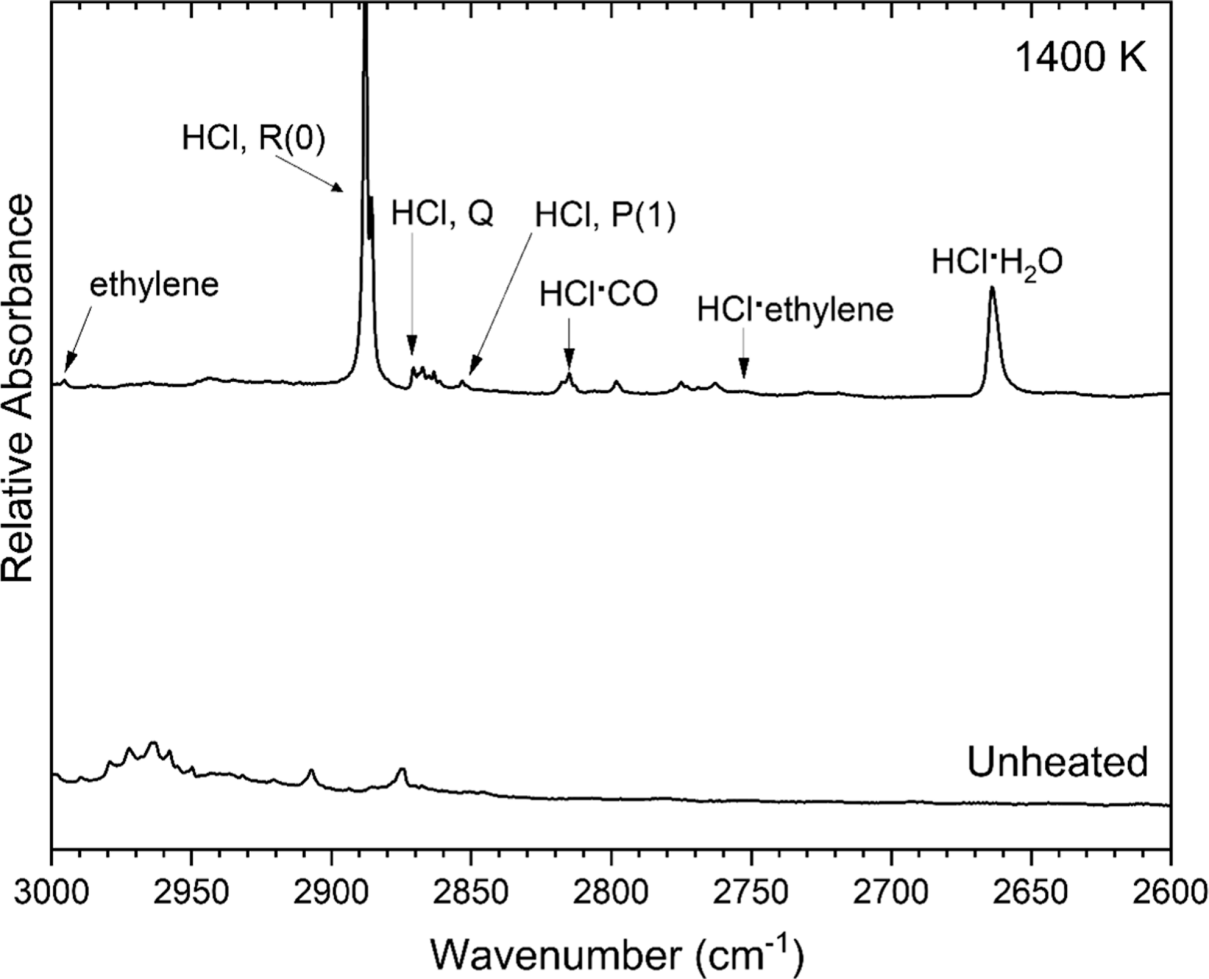
Argon-matrix FTIR spectrum of 0.07% (2-chloroethyl)­benzene (bottom trace) and a spectrum collected following pyrolysis of the sample at 1400 K.

HCl elimination reactions are common in both the pyrolysis and photolysis of many chlorinated hydrocarbons. A four-centered HCl elimination reaction of (2-chloroethyl)­benzene should lead to the formation of styrene. To determine whether styrene was indeed a pyrolysis product, a commercial sample of styrene was obtained, and an argon-matrix FTIR spectrum was recorded. The unheated sample acted as a benchmark to compare whether the bands for styrene matched any peaks in the spectrum from the heated sample of (2-chloroethyl)­benzene. As Figure 4 shows, there is convincing evidence for assigning styrene as a pyrolysis product. The matching peaks are located at 1084, 1022, 998, 992, 979, 909, 903, 778, and 695 cm^–1^. Additional bands of styrene can be seen at 3114, 3100, 3093, 3073, 3054, 3044, 3031, 3023, 1498, 1454, 1318, 1293, 1207, and 1103 cm^–1^ in Figures S2 and S3 of the Supporting Information. These bands are also close to those reported by McMahon and Chapman for styrene, formed by >440 nm irradiation of 1-phenyldiazoethane in a 15 K argon matrix.^40^

**Figure 4:**
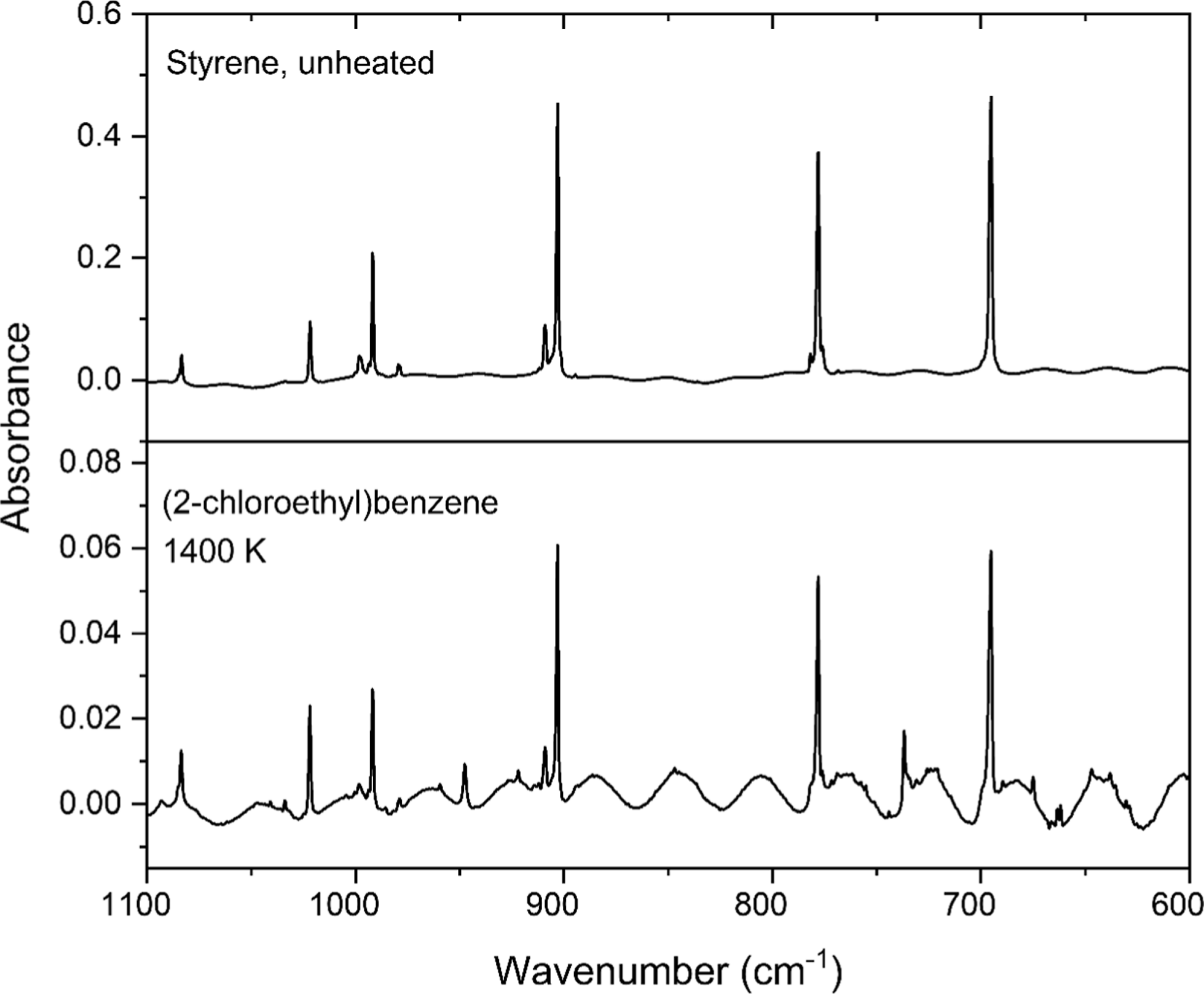
Argon-matrix FTIR spectrum of pyrolyzed 0.07% (2-chloroethyl)­benzene and a benchmark spectrum of 0.1% styrene in argon.

Phenylacetylene was also observed as a pyrolysis product of (2-chloroethyl)­benzene. Assigning this product presented a challenge because some of the bands of phenylacetylene would be expected to overlap those of styrene and those of other possible products that contain an aromatic ring. Furthermore, a quick survey of the experimental spectra showed that phenylacetylene, if present, would have intensities that would be much lower than those observed for styrene. Davis and Andrews^41^ reported a spectrum of phenylacetylene in an argon matrix at 12 K in their study of complexes with HF but did not report wavenumbers for all of the vibrational bands. In order to be certain of the assignment, a commercial sample of phenylacetylene was obtained, and an argon-matrix spectrum was recorded at 4 K for a careful comparison and certainty of the assignments. The highest intensity band of phenylacetylene can be seen at 3340 cm^–1^ in Figure 5, plus several lower absorbance bands in the alkyne C–H stretching region. A band at 3054 cm^–1^ overlaps a band of styrene, which has already been assigned. Additional bands of phenylacetylene can be seen at 758, 755, 689, 647, and 610 cm^–1^ in Figure S4 of the Supporting Information.

**Figure 5:**
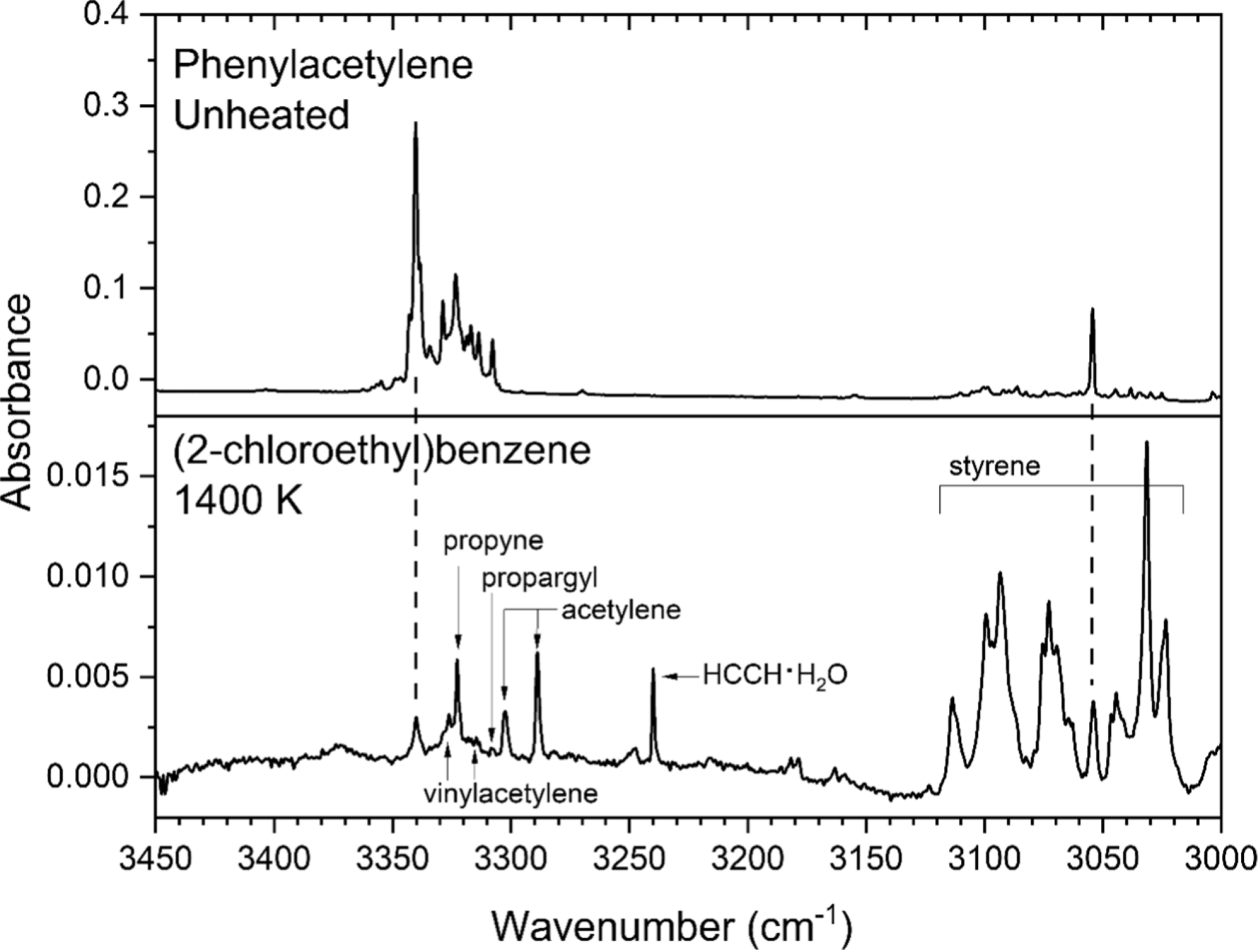
Argon-matrix FTIR spectrum of pyrolyzed 0.07% (2-chloroethyl)­benzene and a benchmark spectrum of 0.1% phenylacetylene in argon.

Benzene formation is suggested by a band at 675 cm^–1^ in Figure 6. This corresponds to a very strong band reported at 677 cm^–1^ by Boganov et al.^42^ Bands observed at 1483 and 1041 cm^–1^ (Figure 7) and at 1956 cm^–1^ (Figure 8) may correspond to strong bands of benzene reported at 1480, 1041, and 1958 cm^–1^, respectively. Other strong bands of benzene reported in the literature at 3098, 3078, and 3044 cm^–1^ overlap bands of styrene and thus could not be discerned here. The bands observed at 675, 1041, and 1483 cm^–1^ are unique, not overlapping those of any other products assigned in these experiments.

**Figure 6:**
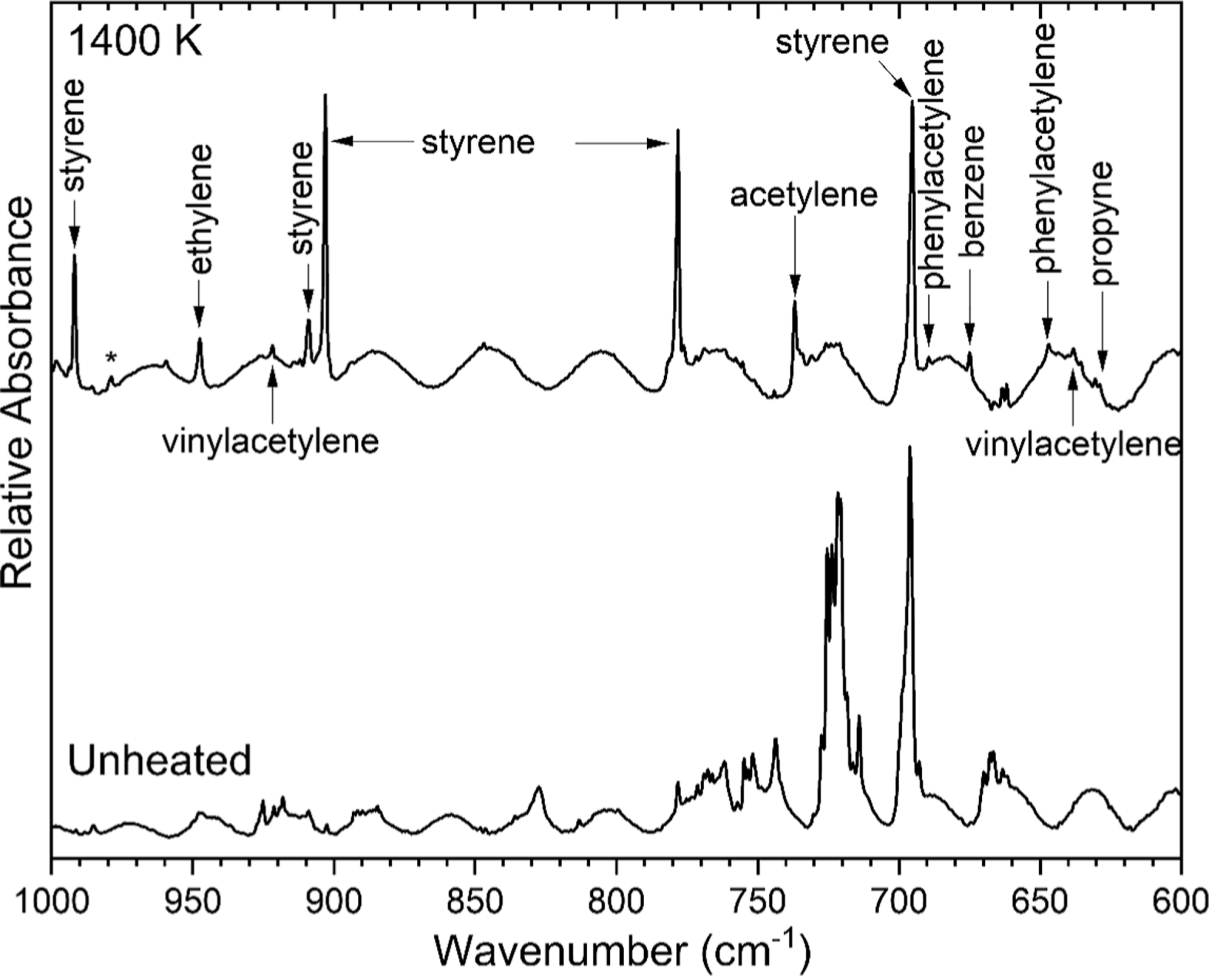
Argon-matrix FTIR spectrum of 0.07% (2-chloroethyl)­benzene (bottom trace) and a spectrum collected following pyrolysis of the sample at 1400 K. The band marked with an asterisk could belong to either vinylacetylene or styrene. Small oscillations in the baseline are due to an etalon effect of a relatively thin argon matrix.

**Figure 7:**
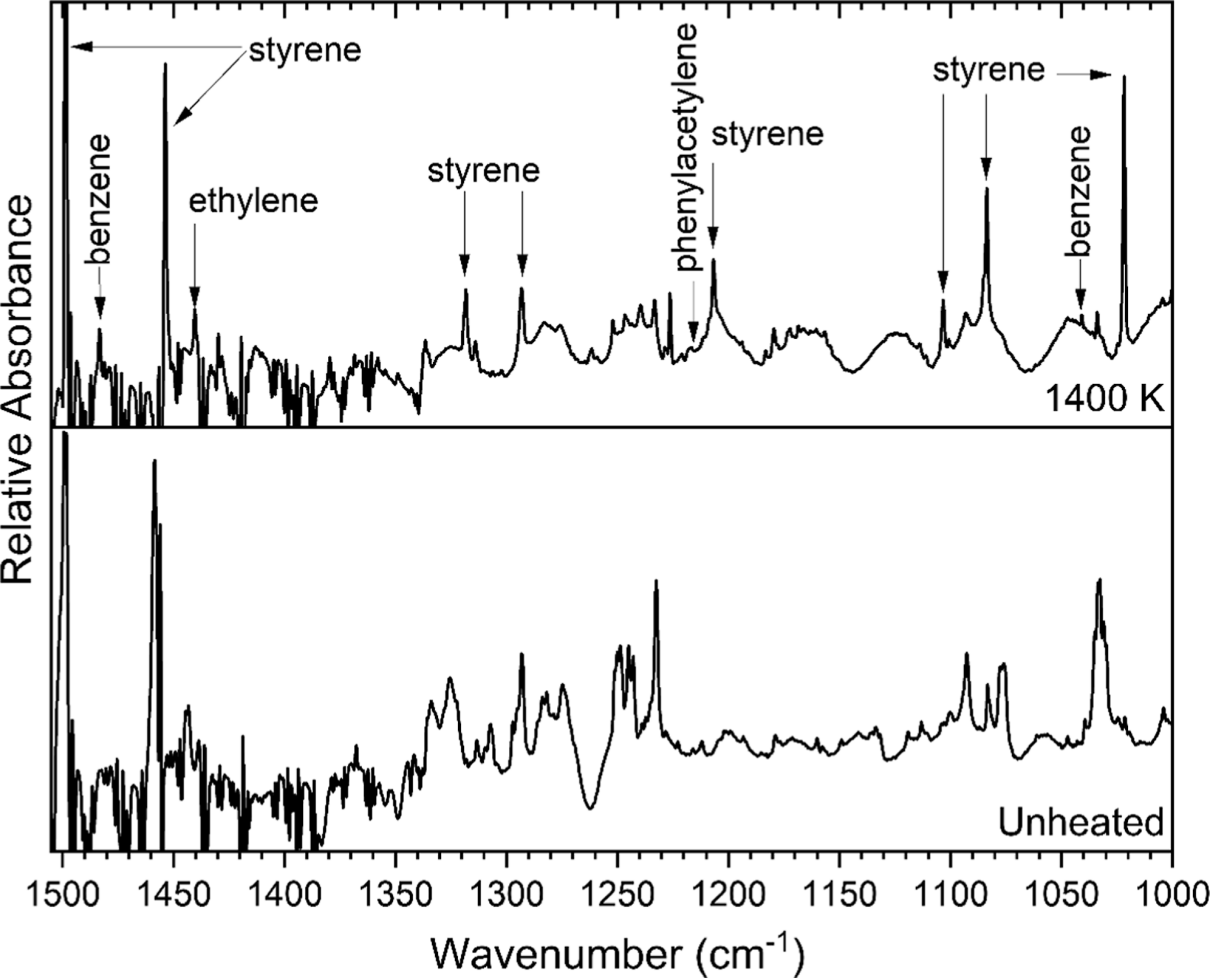
Argon-matrix FTIR spectrum of 0.07% (2-chloroethyl)­benzene (bottom trace) and a spectrum collected following pyrolysis of the sample at 1400 K.

**Figure 8:**
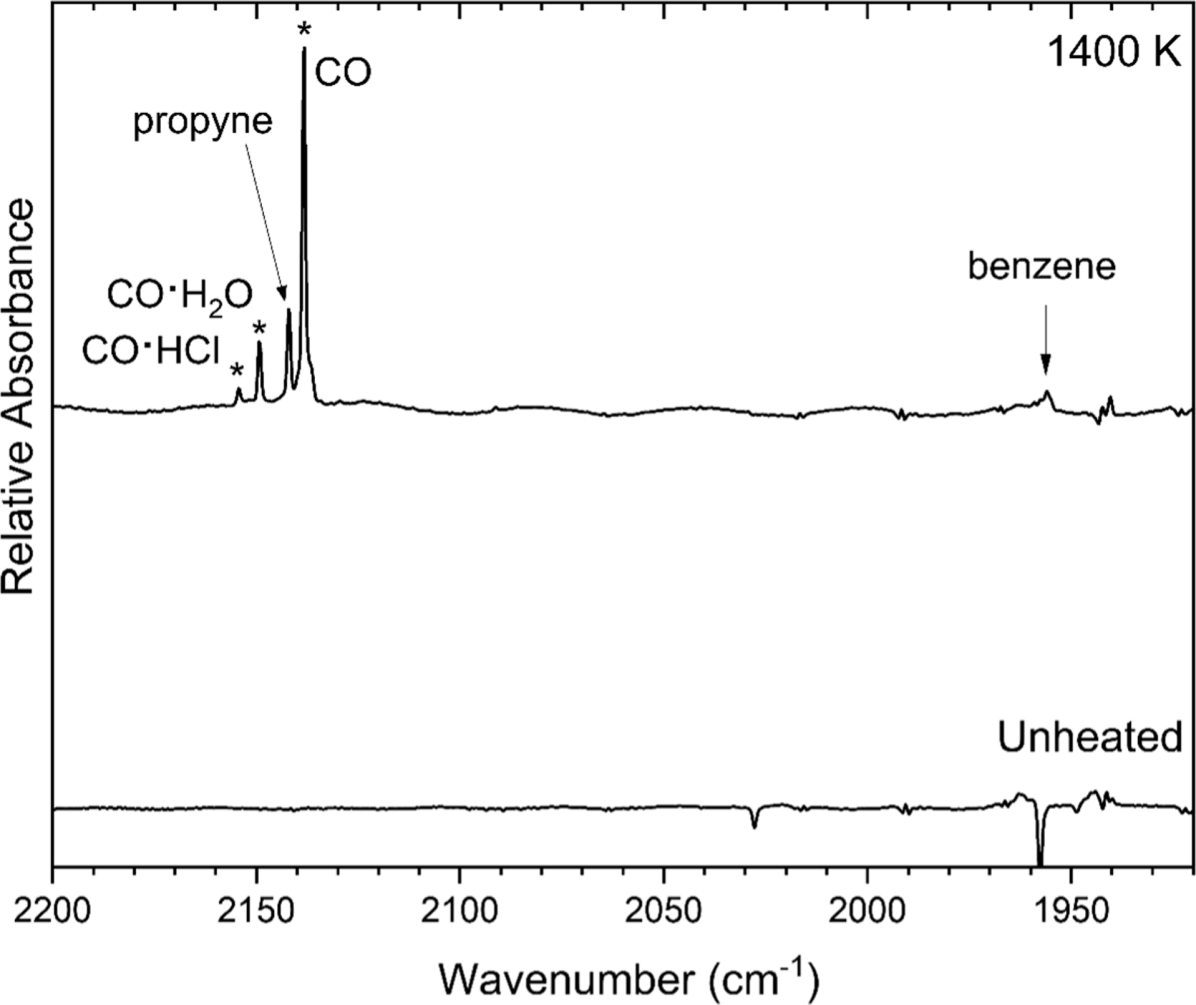
Argon-matrix FTIR spectrum of 0.07% (2-chloroethyl)­benzene (bottom trace) and a spectrum collected following pyrolysis of the sample at 1400 K. The bands marked with an asterisk are CO or CO-cluster contaminants derived from reactions of the hot SiC tube with trace amounts of oxygen. These were also observed in control experiments with heated argon.

Many small hydrocarbon products were observed as pyrolysis products of (2-chloroethyl)­benzene. Vinylacetylene^43,44^ bands were found near 636, 927, 979, 3315, and 3326 cm^–1^ as shown in Figures 6 and 9. Acetylene was evidenced^45^ by bands at 737, 3289, and 3302 cm^–1^ in Figures 6 and 9. A band for the acetylene-water cluster is evident at 3240 cm^–1^.^46^ Propyne^47,48^ was observed at 627 and 2141 cm^–1^ as shown in Figures 6 and 8, respectively, and overlapping a phenylacetylene band at 3323 cm^–1^ in Figure 9. Bands associated with ethylene^49,50^ were seen at 948, 1440, and 2995 cm^–1^ in Figures 6, 7, and 3, respectively. Finally, the propargyl radical^51^ was assigned as a pyrolysis product, with a band observed at 3309 cm^–1^ as shown in Figure 9. The band expected at 687 cm^–1^ for propargyl overlaps a band of phenylacetylene at 689 cm^–1^ as shown in Figure 6. The spectra were examined for another commonly reported band of propargyl at 484 cm^–1^, but it was found to be present in the spectra for both the heated sample and the unheated one. The presence of propargyl is not surprising given the presence of the aromatic ring in (2-chloroethyl)­benzene. The propargyl radical is a known precursor to aromatic ring formation and eventually polycyclic aromatic hydrocarbons and soot.^52–54^

**Figure 9:**
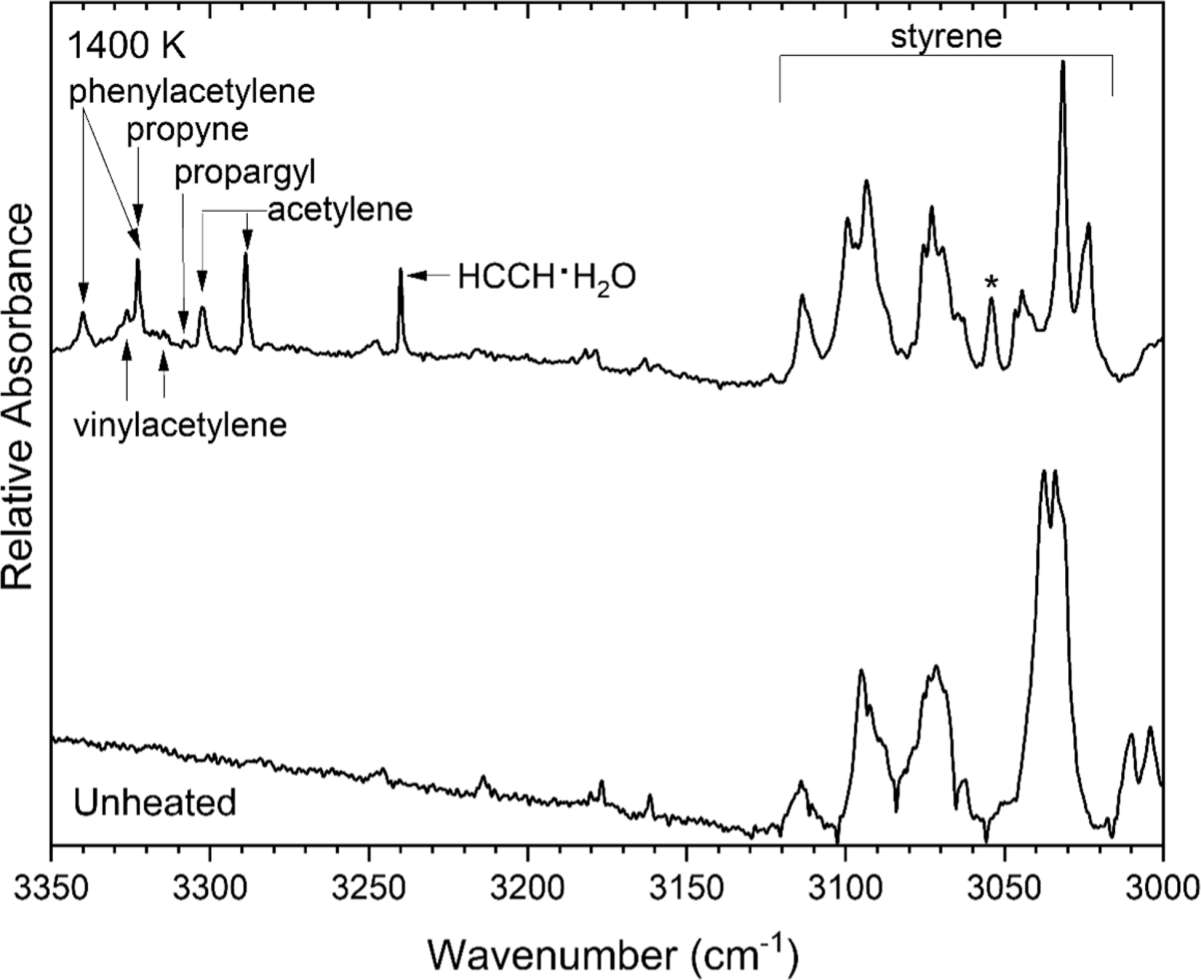
Argon-matrix FTIR spectrum of 0.07% (2-chloroethyl)­benzene (bottom trace) and a spectrum collected following pyrolysis of the sample at 1400 K. The band labeled with an asterisk could belong to both styrene and phenylacetylene.

The products observed here invite questions regarding the pyrolysis mechanism. The presence of the chlorine atom in (2-chloroethyl)­benzene is likely to have a profound influence, which could be seen by a comparison to the pyrolysis of its non-chlorinated analogue ethylbenzene. Early studies of ethylbenzene pyrolysis led to some debate over the mechanism, as some experiments conducted at temperatures below 1000 °C suggested an initial step forming the benzyl radical plus methyl radical^55^ while an early shock tube study at 1250–1600 K suggested H loss from the 1-ethyl position as the initial step with a final product of styrene.^56^ Transition state theory calculations show that methyl loss is the lowest energy and exclusively dominant channel for 800–2000 K pyrolysis.^57^ Here, there is no evidence of the production of chloromethyl radicals or benzyl radicals following the pyrolysis of (2-chloroethyl)­benzene. Another helpful analogue to consider for insight into the pyrolysis of (2-chloroethyl)­benzene is 1-bromo-1-phenylethane. During pyrolysis, 1-bromo-1-phenylethane decomposes via HBr elimination to make styrene.^58^ The evidence for styrene and HCl production from (2-chloroethyl)­benzene observed here is quite convincing, but the eventual fate of styrene is an interesting point to consider. Styrene pyrolysis is known to lead to polycyclic aromatic hydrocarbon production via bimolecular reactions, as observed for high-pressure samples^59^ at 1100–1730 K and the liquid^60^ at 500–900 °C. A low-pressure (∼10 mTorr) flow reactor study of styrene at 1180–1350 K detected benzene and acetylene and suggested a unimolecular pathway.^61^ These latter conditions are more relevant to the experiments conducted here and suggest that benzene and acetylene observed in the pyrolysis of (2-chloroethyl)­benzene could be produced by reactions of styrene.

To better understand the mechanism of the formation of styrene and then phenylacetylene, computational chemistry was used to map the possible pathways to these products. Figure 10 shows the B3LYP-optimized geometries of the *anti-* and *gauche*-conformers of (2-chloroethyl)­benzene, styrene, phenylacetylene, intermediates, and transition states along those paths. Figure 11 shows the routes to the formation of phenylacetylene via styrene through molecular elimination reactions. Figure 12 shows the routes to the formation of these products through single-bond scission reactions. These results show the molecular elimination pathway to be much lower in energy.

**Figure 10:**
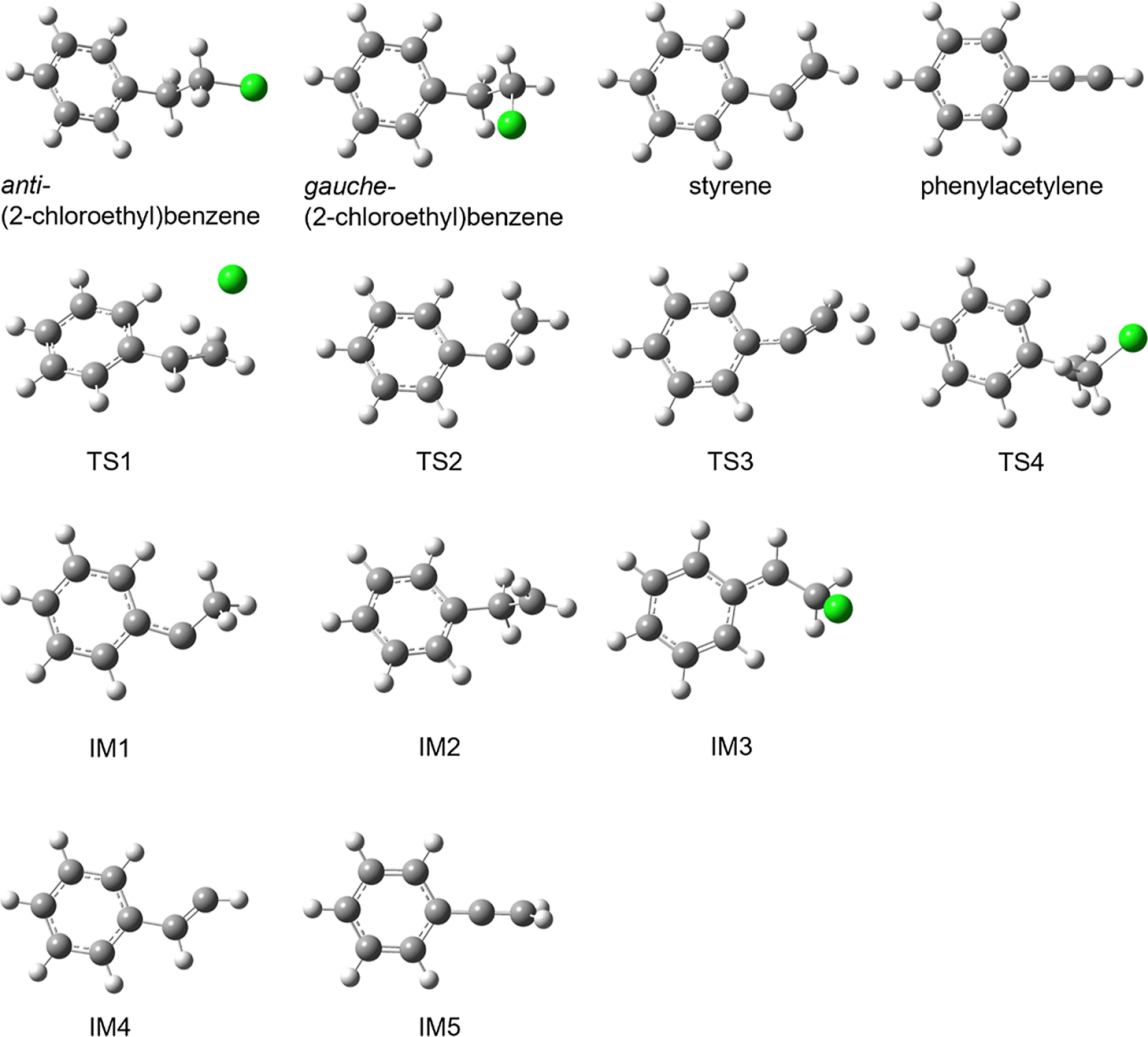
Structures of species, referenced in Figures 11 and 12, involved in the reactions of (2-chloroethyl)­benzene forming styrene and phenylacetylene, optimized at the B3LYP/6-311++G­(d,p) level of theory.

**Figure 11:**
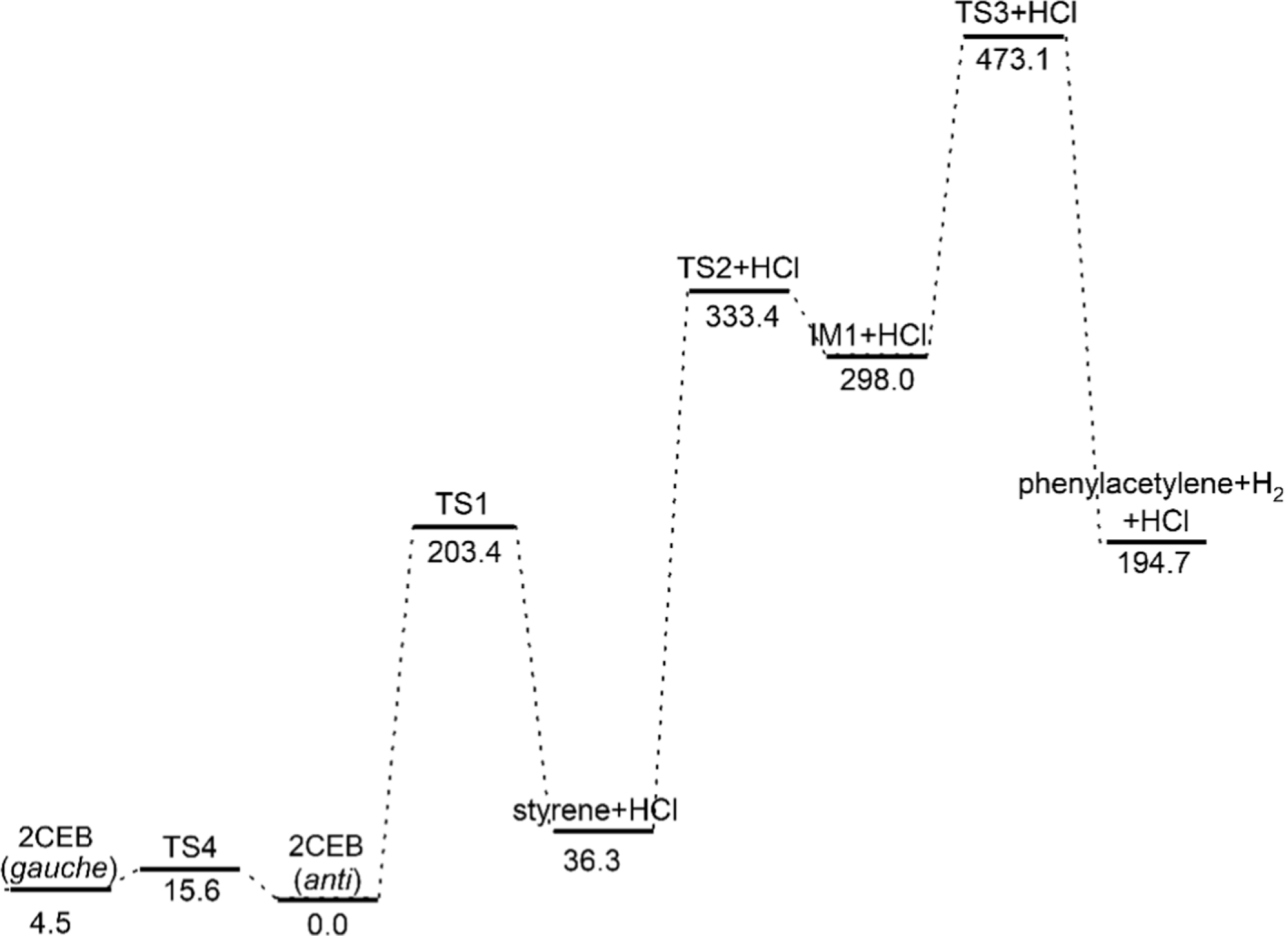
Pathway of (2-chloroethyl)­benzene (2CEB) forming phenylacetylene via molecular elimination mechanisms with zero-point corrected energies in kJ/mol, relative to the energy of *anti*-(2-chloroethyl)­benzene. The *anti*- to *gauche*-isomerization is shown as well.

**Figure 12:**
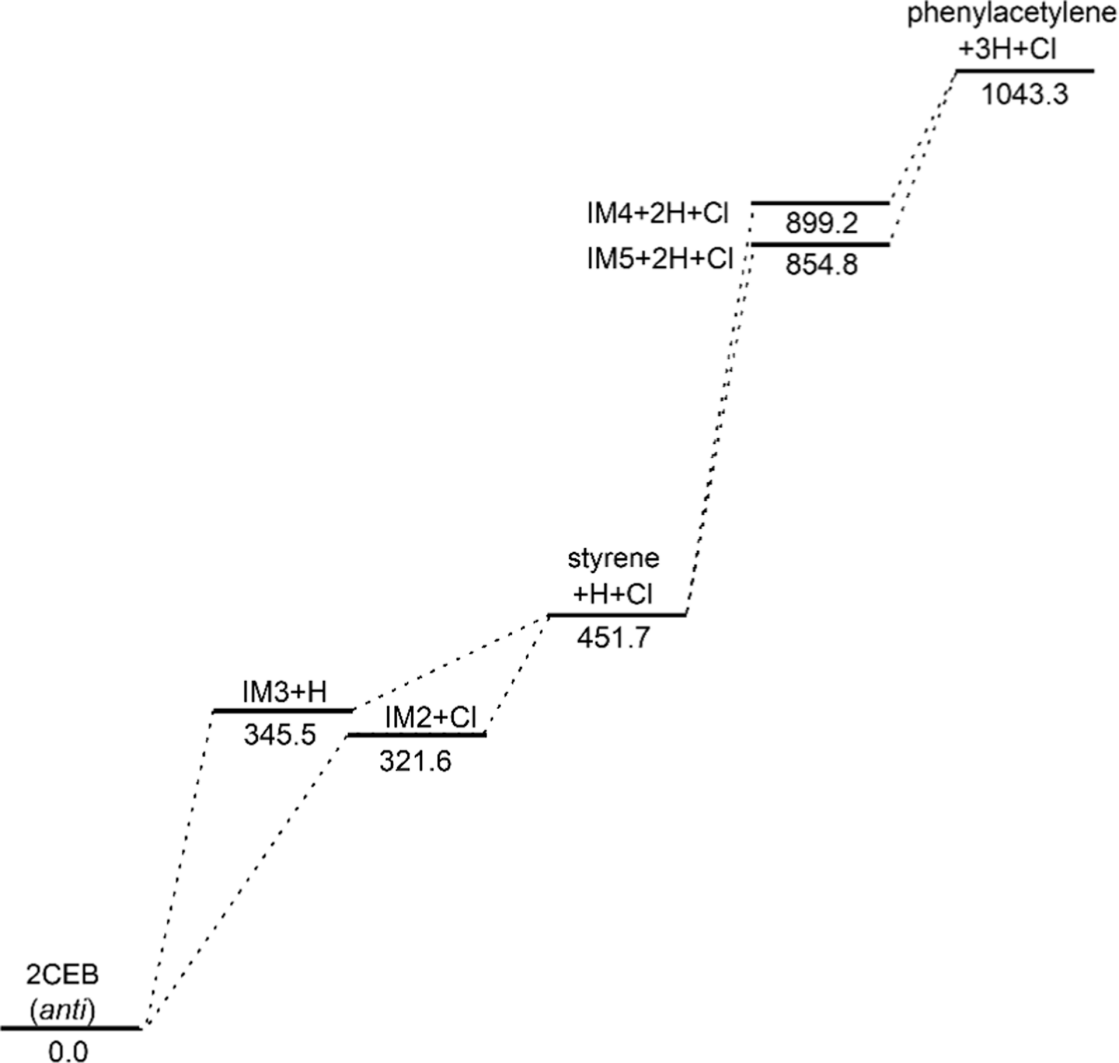
Pathway of (2-chloroethyl)­benzene (2CEB) forming phenylacetylene via single-bond scission mechanisms with zero-point corrected energies in kJ/mol, relative to the energy of *anti*-(2-chloroethyl)­benzene.

## Conclusions

The pyrolysis products of (2-chloroethyl)­benzene in argon were identified by matrix-isolation FTIR spectroscopy. It was observed that the pyrolysis products of (2-chloroethyl)­benzene were HCl, styrene, phenylacetylene, benzene, vinylacetylene, acetylene, propyne, ethylene, propargyl radical, and several HCl-based complexes. Styrene was a predominant product likely produced through a four-centered elimination mechanism. Other products, particularly phenylacetylene, benzene, and acetylene, may be due to the secondary reactions of styrene. These results provide a good foundation for mechanism development and have potential applications in the management of chlorinated hydrocarbons generated during PVC chemical recycling.

## Supplementary Material



## Notes

Argon-matrix FTIR spectra showing possible clusters of HCl with phenylacetylene, styrene, or water; additional spectra supporting the assignments of styrene and phenylacetylene as pyrolysis products; and Cartesian coordinates of B3LYP/6-311++G­(d,p) optimized structures for species involved in the reactions forming styrene and phenylacetylene (PDF)
