# Supplementary material for: Pyrolysis Reactions of (2-Chloroethyl)benzene
Source: J Phys Chem A. 2025 Oct 3;129(41):9521–8. doi: 10.1021/acs.jpca.5c03721 (PMC12536402; doi:10.1021/acs.jpca.5c03721)
Supplement: Supplementary file 1 [file jp5c03721_si_001.pdf]

Supporting Information
Pyrolysis Reactions of (2-Chloroethyl)benzene

Mia Jarrell, Tess Courtney, Khaled El-Shazly, David Kapp, Drew Fields, Alexis Bowles, Laura R. McCunn

Department of Chemistry, Marshall University

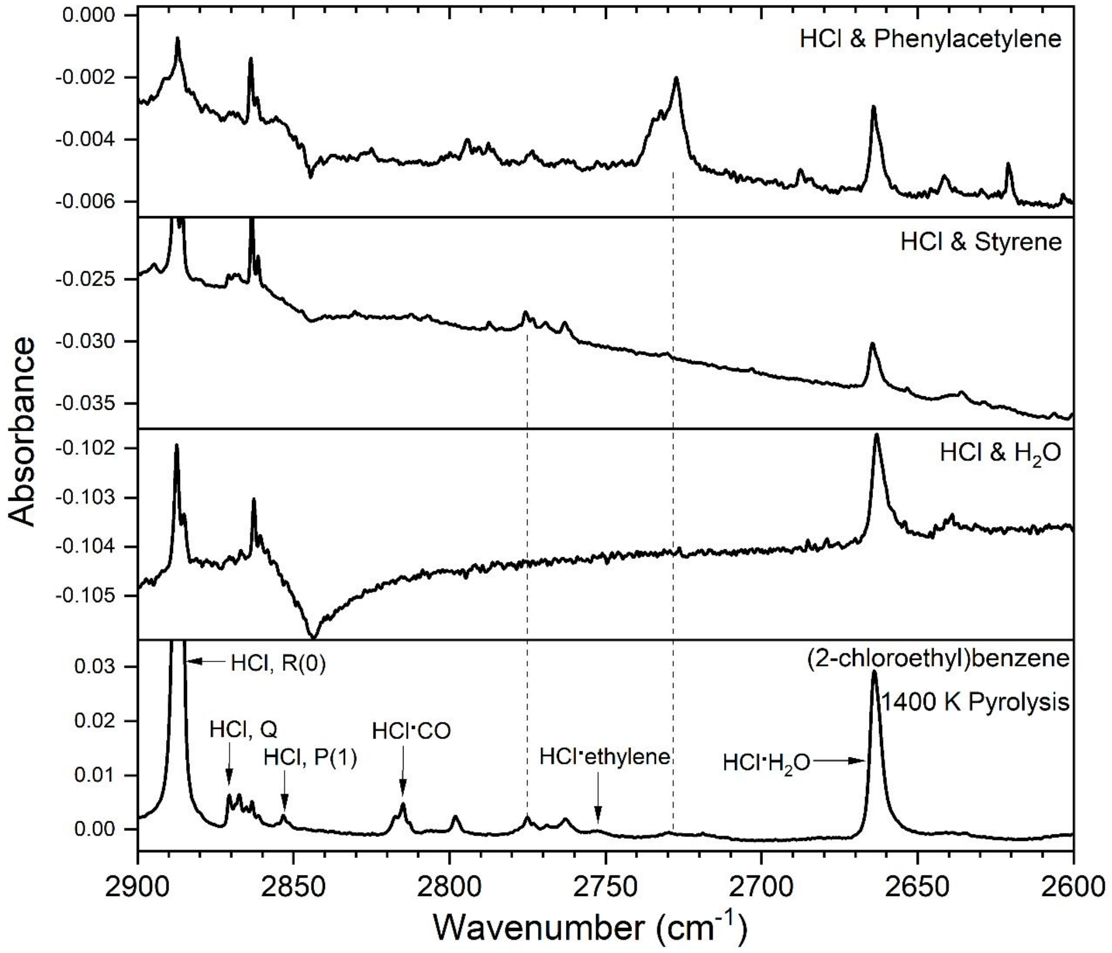

Figure S1. Argon-matrix FTIR spectra of various samples codeposited with HCl in a 1:1:1000 (sample:HCl:Ar) ratio. These are shown above the spectrum collected following the 1400 K pyrolysis of 0.07% (2-chloroethyl)benzene to demonstrate that the peaks in the 2850-2700 cm^{-1} may be partly due to clusters of styrene·HCl but are unlikely to be phenylacetylene·HCl.

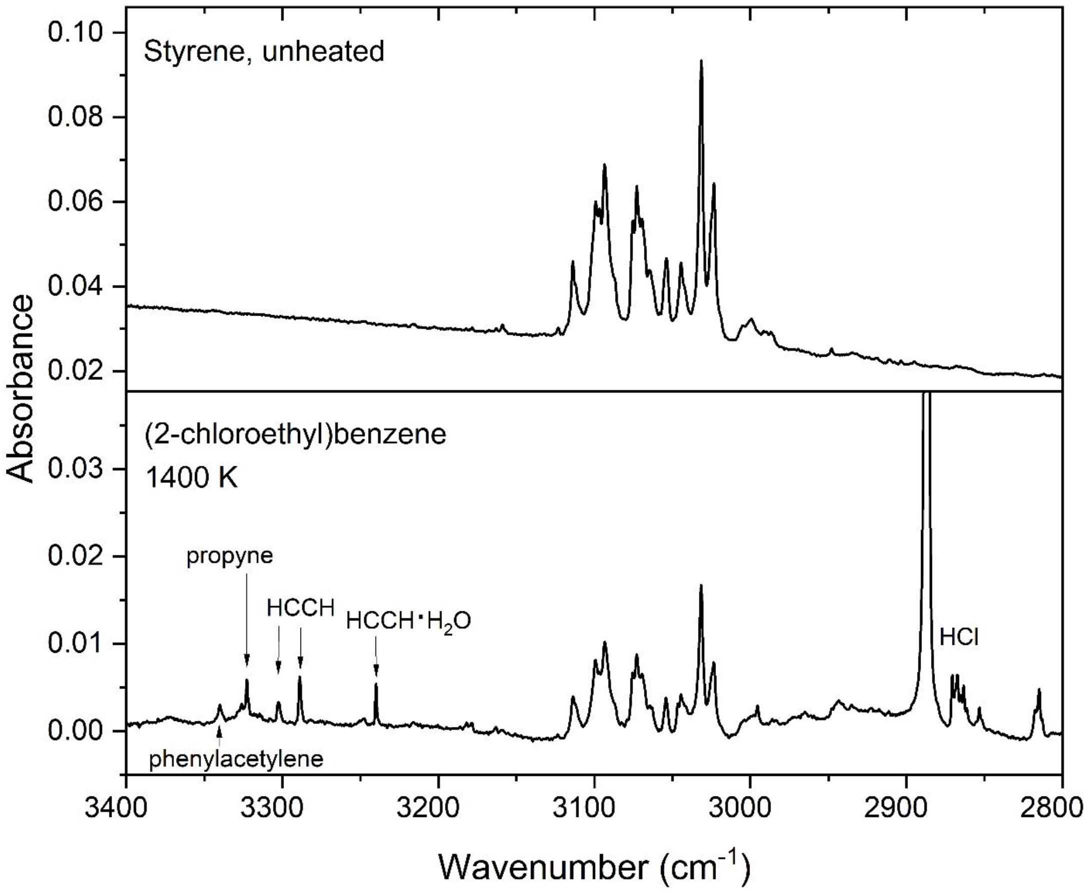

Figure S2. Spectrum of matrix-isolated styrene (0.1% in argon) compared to a spectrum collected following the 1400 K pyrolysis of 0.07% (2-chloroethyl)benzene.

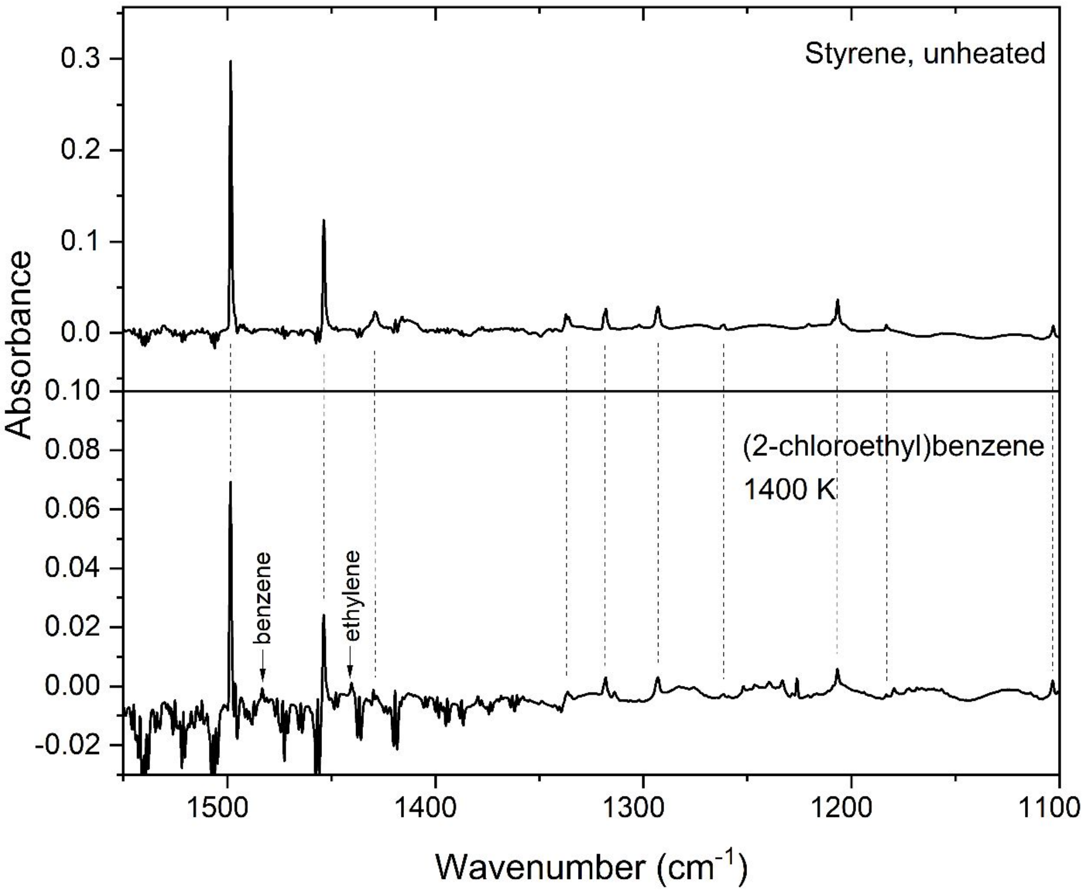

Figure S3. Spectrum of matrix-isolated styrene (0.1% in argon) compared to a spectrum collected following the 1400 K pyrolysis of 0.07% (2-chloroethyl)benzene.

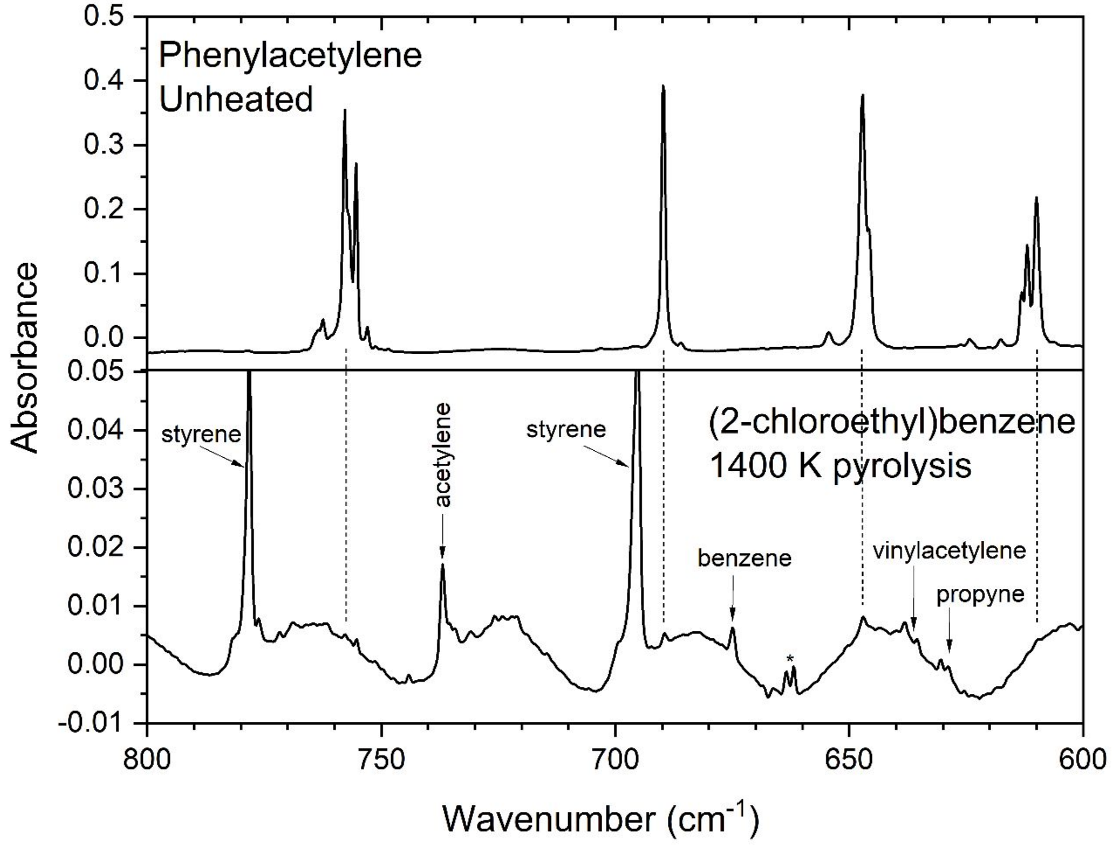

Figure S4. Spectrum of matrix-isolated phenylacetylene (0.1% in argon) compared to a spectrum collected following the 1400 K pyrolysis of 0.07% (2-chloroethyl)benzene.

Cartesian Coordinates from B3LYP/6-311++G(d,p) Optimization and Frequency Calculations

***anti*-(2-chloroethyl)benzene**

Symbol	X	Y	Z
C	3.2870600	-0.0000210	-0.3244650
C	2.6090270	1.2045840	-0.1457470
C	1.2609940	1.2023200	0.2070870
C	0.5681760	0.0000230	0.3881560
C	1.2609860	-1.2022930	0.2072130
C	2.6090220	-1.2046020	-0.1456210
H	4.3364810	-0.0000350	-0.5964800
H	3.1304890	2.1463690	-0.2760390
H	0.7426260	2.1451770	0.3522030
H	0.7426190	-2.1451360	0.3524270
H	3.1304740	-2.1464070	-0.2758140
C	-0.9057680	0.0000480	0.7361900
C	-1.7607870	-0.0000430	-0.5301580
H	-1.1544610	0.8800140	1.3350210
H	-1.1544620	-0.8798340	1.3351450
H	-1.5844000	0.8874790	-1.1354550
H	-1.5844090	-0.8876580	-1.1353210
Cl	-3.5398360	-0.0000040	-0.1359770

TS1: Transition State for HCl loss from (2-chloroethyl)benzene to form styrene

Symbol	X	Y	Z
C	-3.0544940	-0.7327830	-0.0821390
C	-2.9122210	0.6124060	-0.4169820
C	-1.6609810	1.2200390	-0.3509470
C	-0.5378800	0.4942600	0.0633190
C	-0.6857220	-0.8624560	0.3821860
C	-1.9367320	-1.4677890	0.3122900
H	-4.0269120	-1.2082960	-0.1377650
H	-3.7739070	1.1895060	-0.7325460
H	-1.5558210	2.2671310	-0.6153570
H	0.1810840	-1.4562110	0.6512220
H	-2.0362550	-2.5196120	0.5547640
C	0.7823970	1.1769370	0.1345270
C	1.7664230	0.8686670	1.0850420
H	0.8441000	2.1642770	-0.3169490
H	1.6286090	0.4946340	-0.5035040

H	2.6403660	1.4940190	1.2011840
H	1.6760680	0.0220980	1.7526750
Cl	3.1681130	-0.6060720	-0.5069110

HCl

Symbol	X	Y	Z
Cl	0.0000000	0.0000000	0.0715020
H	0.0000000	0.0000000	-1.2155360

Styrene

Symbol	X	Y	Z
C	-2.2623690	0.2615750	0.0000230
C	-1.3599990	1.3276710	-0.0000150
C	0.0090110	1.0905530	-0.0000430
C	0.5141470	-0.2205760	-0.0000330
C	-0.4061740	-1.2798070	0.0000040
C	-1.7790690	-1.0443620	0.0000350
H	-3.3299290	0.4501330	0.0000390
H	-1.7278730	2.3478370	-0.0000290
H	0.6913110	1.9325230	-0.0000750
H	-0.0364840	-2.3002600	0.0000150
H	-2.4693520	-1.8806100	0.0000640
C	1.9534760	-0.5288470	-0.0000540
H	2.1863680	-1.5915630	-0.0001870
C	2.9733780	0.3351800	0.0000720
H	2.8336860	1.4104340	0.0002160
H	3.9978780	-0.0168130	0.0000290

TS2: Transition State for hydrogen migration in styrene to form c-C_6H_5-CCH_3

Symbol	X	Y	Z
C	-2.2347640	0.2356810	0.1434590
C	-1.3665980	1.3121820	-0.0353950
C	0.0000750	1.0922150	-0.1826750
C	0.5218250	-0.2157240	-0.1946680
C	-0.3742090	-1.2920200	-0.0613980
C	-1.7311580	-1.0668050	0.1402540
H	-3.2976610	0.4084010	0.2691770
H	-1.7543040	2.3250760	-0.0504340
H	0.6677960	1.9365300	-0.3191290
H	0.0134240	-2.3030490	-0.1174390

H	-2.4041060	-1.9078420	0.2662870
C	1.9447520	-0.4638250	-0.4409740
H	2.6381750	-0.9422770	0.6135070
C	2.8494430	0.2715110	0.3259860
H	2.5631270	1.0539090	1.0453420
H	3.9173550	0.1899680	0.1251570

IM1: c-C_6H_5-CCH_3

Symbol	X	Y	Z
C	-2.2357910	0.3119900	0.1034220
C	-1.3099110	1.3485540	-0.0425260
C	0.0455580	1.0618180	-0.1264920
C	0.5055630	-0.2796990	-0.1347190
C	-0.4618410	-1.3099880	-0.0456030
C	-1.8087490	-1.0179960	0.1190660
H	-3.2923450	0.5415670	0.1891570
H	-1.6517170	2.3773870	-0.0726540
H	0.7557740	1.8739570	-0.2254350
H	-0.1111280	-2.3338700	-0.0993410
H	-2.5326930	-1.8187890	0.2186750
C	1.8818690	-0.6778880	-0.2963270
H	2.6295790	1.2788770	0.4803550
C	2.9107760	0.2456680	0.2162460
H	3.8129910	0.2342150	-0.4029030
H	3.2246870	-0.2481000	1.1537470

TS3: Transition State for H_2 elimination from c-C_6H_5-CCH_3 to form phenylacetylene + H_2

Symbol	X	Y	Z
C	2.3035490	0.1816700	0.1145570
C	1.7508030	-1.0993330	0.1135510
C	0.3813150	-1.2764850	-0.0523010
C	-0.4833400	-0.1681250	-0.1513870
C	0.0895350	1.1221540	-0.1353630
C	1.4656760	1.2887000	-0.0279130
H	3.3745680	0.3157540	0.2143330
H	2.3940320	-1.9671000	0.2123180
H	-0.0401650	-2.2735780	-0.1015300
H	-0.5551450	1.9890690	-0.2286670
H	1.8855200	2.2889660	-0.0360480
C	-1.9027570	-0.3737740	-0.3089300
H	-3.1932030	0.8604070	1.0946280
C	-2.8912410	0.1903620	0.3023570

H	-4.0891120	-0.5224490	0.1439100
H	-4.0577380	0.1179200	-0.4263660

H_2

Symbol	X	Y	Z
H	0.0000000	0.0000000	0.3720760
H	0.0000000	0.0000000	- 0.3720760

Phenylacetylene

Symbol	X	Y	Z
C	-2.2096280	0.0000490	-0.0000080
C	-1.5100400	-1.2065290	0.0000320
C	-0.1193090	-1.2109280	-0.0000280
C	0.5938080	-0.0000680	-0.0000330
C	-0.1192300	1.2108860	-0.0000030
C	-1.5099320	1.2066000	0.0000090
H	-3.2936900	0.0001060	0.0000100
H	-2.0491460	-2.1471300	0.0000480
H	0.4267840	-2.1464930	-0.0000120
H	0.4269890	2.1463760	0.0000040
H	-2.0489940	2.1472250	0.0000210
C	2.0220280	-0.0000580	-0.0000170
C	3.2270530	0.0000300	0.0000270
H	4.2895500	0.0000240	0.0000540

TS4: Transition State for *anti*- to *gauche*- isomerization of (2-chloroethyl)benzene

Symbol	X	Y	Z
C	3.1260380	0.5174940	-0.3024280
C	2.1438670	1.4537350	0.0207000
C	0.8587320	1.0335100	0.3547370
C	0.5307030	-0.3279700	0.3699800
C	1.5249920	-1.2567030	0.0475310
C	2.8132600	-0.8397150	-0.2870190
H	4.1274790	0.8439570	-0.5590370
H	2.3805550	2.5119930	0.0181500
H	0.1020800	1.7685400	0.6101150
H	1.2916900	-2.3168360	0.0633970
H	3.5715560	-1.5755560	-0.5308950
C	-0.8758440	-0.7775160	0.7039470

C	-1.8492720	-0.6575150	-0.4911180
H	-1.2632850	-0.1898840	1.5384230
H	-0.8573060	-1.8174910	1.0402810
H	-1.3601080	-0.2441690	-1.3698150
H	-2.2991060	-1.6134460	-0.7501090
Cl	-3.2546130	0.4565280	-0.1505000

***gauche*-(2-chloroethyl)benzene**

Symbol	X	Y	Z
C	2.8711910	-0.5060170	-0.4068100
C	2.4734540	0.8045000	-0.6675510
C	1.2378840	1.2614130	-0.2144750
C	0.3780930	0.4200320	0.5010660
C	0.7896070	-0.8911050	0.7581160
C	2.0258340	-1.3518810	0.3083320
H	3.8339580	-0.8630830	-0.7546840
H	3.1276610	1.4726930	-1.2166190
H	0.9421880	2.2875600	-0.4127220
H	0.1355660	-1.5568500	1.3104450
H	2.3281980	-2.3719990	0.5175490
C	-0.9670760	0.9240180	0.9757920
C	-2.0722150	0.9010240	-0.0794260
H	-0.8701620	1.9692350	1.2946650
H	-1.2986000	0.3584980	1.8504650
H	-2.9728370	1.3913000	0.2867930
H	-1.7542200	1.3709650	-1.0087930
Cl	-2.5819050	-0.7900130	-0.5245510

IM2: c-C_6H_5-CH_2CH_2

Symbol	X	Y	Z
C	-0.3214250	1.2498490	-0.0722550
C	-1.6926470	1.1214960	0.1519160
C	-2.2795120	-0.1410180	0.1872150
C	-1.4865400	-1.2732310	-0.0024140
C	-0.1181260	-1.1400900	-0.2234550
C	0.4837080	0.1236690	-0.2622280
H	0.1278000	2.2376770	-0.1008980
H	-2.3002200	2.0080710	0.2968340
H	-3.3451180	-0.2436470	0.3588840
H	-1.9355040	-2.2602580	0.0199850
H	0.4929100	-2.0254250	-0.3682140
C	1.9771060	0.2554780	-0.4890770

H	2.2639850	-0.2976040	-1.3934130
H	2.2076340	1.3090980	-0.7182170
C	2.8074580	-0.2099300	0.6623270
H	3.8520810	-0.4608730	0.5210930
H	2.4162980	-0.1843710	1.6717760

IM3: c-C_6H_5-$CHCH_2Cl$

Symbol	X	Y	Z
C	3.0263930	-0.6038190	-0.1999370
C	1.9720420	-1.4378820	0.1873110
C	0.7021320	-0.9227320	0.3884760
C	0.4395990	0.4628300	0.2075490
C	1.5264440	1.2872030	-0.1956320
C	2.7919360	0.7625590	-0.3913070
H	4.0172700	-1.0143550	-0.3554050
H	2.1473150	-2.4989090	0.3259170
H	-0.1026550	-1.5912820	0.6684150
H	1.3490520	2.3466990	-0.3470870
H	3.6038440	1.4132980	-0.6961920
C	-0.8379450	1.0428030	0.4114830
C	-2.0378060	0.3247000	0.8542270
H	-0.9586000	2.0924350	0.1644120
H	-1.8363270	-0.5373280	1.4842510
H	-2.7598150	0.9780110	1.3361710
Cl	-2.9974620	-0.3930910	-0.5972650

IM4: c-C_6H_5-CH-CH

Symbol	X	Y	Z
C	-2.2107040	0.2416360	-0.0000020
C	-1.3255790	1.3226200	0.0000410
C	0.0463960	1.1062890	0.0000190
C	0.5648340	-0.1987320	-0.0000190
C	-0.3328020	-1.2747880	-0.0000810
C	-1.7091130	-1.0575480	-0.0000130
H	-3.2810960	0.4135740	-0.0000270
H	-1.7097020	2.3367740	0.0000300
H	0.7311820	1.9470040	0.0000130
H	0.0524710	-2.2893940	-0.0000740
H	-2.3879030	-1.9031290	-0.0000320
C	2.0154060	-0.4614330	0.0001710
H	2.2940860	-1.5223470	0.0003930
C	2.9902300	0.4208970	-0.0001550

H	4.0689460	0.4238700	-0.0000640

IM5: c-C_6H_5-C-CH_2

Symbol	X	Y	Z
C	2.2790070	0.0001750	0.0001890
C	1.5725790	1.2110450	0.0000670
C	0.1910860	1.2261070	-0.0001610
C	-0.5509880	-0.0002070	-0.0002610
C	0.1913520	-1.2262670	-0.0001290
C	1.5728640	-1.2108370	0.0001020
H	3.3624130	0.0002750	0.0003280
H	2.1145950	2.1504780	0.0002140
H	-0.3513320	2.1637130	-0.0002650
H	-0.3507400	-2.1640640	-0.0001870
H	2.1151160	-2.1501350	0.0002210
C	-1.9199500	-0.0001630	-0.0005810
C	-3.2180550	0.0000690	0.0004240
H	-3.7994430	-0.0002640	-0.9233320
H	-3.7979800	0.0004640	0.9251090
